# Paediatric multicystic dysplastic kidney disease in Cape Town, South Africa

**DOI:** 10.1186/s12882-025-04667-2

**Published:** 2025-11-29

**Authors:** Datonye Christopher Briggs, Khanyisile Hlongwa, Mignon McCulloch, Peter Nourse, Anita Brink, Ashton Coetzee

**Affiliations:** 1 Department of Paediatrics and Child Health, University of Cape Town/ Red Cross War Memorial Children’s Hospital, Cape Town, South Africa; 2 Department of Nuclear Medicine, University of Cape Town/ Red Cross War Memorial Children’s Hospital, Cape Town, South Africa; 3 Department of Nuclear Medicine Sciences Applications, Division of Human Health, Nuclear Medicine and Diagnostic Imaging Section, International Atomic Energy Agency, Vienna, Austria; 4 Department of Paediatrics and Child Health, Rivers State University, Teaching Hospital/ Rivers State University, Port Harcourt, Nigeria

**Keywords:** Chronic kidney disease, Multicystic dysplastic kidney, Paediatrics, Epidemiology, Solitary kidney

## Abstract

**Introduction:**

Multicystic dysplastic kidney disease (MCDK) is a notable congenital anomaly of the kidney and urinary tract, with potential risk for chronic kidney disease, yet data from sub-Saharan Africa remain scarce. This study examined the pattern of MCDK, associated contralateral kidney abnormalities, determined the predictors of MCDK involution and assessed short-term outcomes in children followed beyond one year in South Africa.

**Method:**

This retrospective study involved children under 13 years of age with suspected unilateral MCDK, confirmed on kidney ultrasound and [^99m^Tc]Tc-MAG3 scans at the Red Cross War Memorial Children’s Hospital between January 1, 2014, and December 31, 2023. Demographic, clinical, and radiologic data were obtained. The Log-rank test and Cox Proportional Hazards regression analyses were used to identify predictors of MCDK involution.

**Results:**

Among 1,581 new cases, 98 (6.2%) had unilateral MCDK. 50% were male, and 57.1% had left-sided involvement. Median follow-up was 60 months (IQR: 12–72). Contralateral kidney abnormalities occurred in 17 (17.3%), most commonly duplex kidney (35.3%) and ureteropelvic junction obstruction (29.4%), but no vesicoureteric reflux was noted. Of 81 children followed beyond a year, 80.2% demonstrated contralateral hypertrophy, and 69.1% exhibited involution of the affected kidney. Initial kidney size ≤ 5.0 cm was the sole predictor of involution (Hazard Ratio: 2.42, 95% CI: 1.31–4.48). Urinary tract infections occurred in 18.5%, proteinuria in 2.5%, hypertension in 1.2%, and 2.5% developed chronic kidney disease related to contralateral dysplasia. One nephrectomy was performed, and no malignancies or deaths. At last follow-up, 28.4% were lost to follow-up, and 12.3% had transitioned to adolescent clinics.

**Conclusion:**

All MCDK cases were unilateral, with duplex kidney being the most common contralateral abnormality, a distinctive finding previously unreported. Follow-up into adolescence may be beneficial, as progression of chronic kidney disease is rare in those without contralateral anomalies. Multicentre long-term studies are needed to provide standardised follow-up guidelines.

## Introduction

Multicystic dysplastic kidney disease (MCDK) is a significant congenital anomaly of kidney development, characterised by multiple non-communicating cysts of varying sizes within a non-functioning parenchyma [1]. This genitourinary malformation is frequently identified in utero or during the early neonatal period, with an incidence of approximately 1 in 2,200 to 4,300 live births [2, 3]. MCDK arises from defective and incomplete nephron formation resulting from maldevelopment of the ureteric bud during early kidney morphogenesis [4]. It is one of the leading causes of a palpable abdominal mass in infants, second only to hydronephrosis due to ureteropelvic junction obstruction (UPJO) [4]. While MCDK typically affects a single kidney, bilateral involvement is rare but invariably fatal, as it is associated with kidney failure and pulmonary hypoplasia [4].

Studies suggest that involution of the multicystic dysplastic kidney is most pronounced within the first two to three years of life [5, 6], with approximately 10% occurring by the first year, 35.0% by the second year, and between 38.5% and 47% by the fifth year [4, 7]. Nonetheless, concerns persist regarding an elevated risk of kidney failure in children with MCDK, particularly among those lacking compensatory hypertrophy of the contralateral kidney [5]. Furthermore, up to one-third of affected children may exhibit abnormalities in the solitary functioning contralateral kidney [3], such as vesicoureteral reflux (VUR) [8], ureteropelvic junction obstruction, dysplasia, or positional anomalies, all of which increase the risk of chronic kidney disease [9–11]. Although less frequently reported, hypertension develops in 3.2% of cases [12], urinary tract infections in 5.0%–34.7% [13–16], and malignant transformation in 0.07% [12].

The epidemiology and outcomes of paediatric MCDK in sub-Saharan Africa remain largely underreported. The existing literature is predominantly limited to case reports or small-scale studies [17–20], which hinders a comprehensive understanding of MCDK in this setting. Furthermore, an important aspect of MCDK management is determining the appropriate frequency of ultrasonographic surveillance, which currently lacks standardisation [21, 22]. A recent Canadian study recommended scheduled ultrasounds for children with congenital solitary kidney, including MCDK, at specific intervals (birth, 6 months, 2, 5, 10, and 15 years) to minimise unnecessary imaging and optimise resource utilisation [23]; however, it remains unclear what occurs in Africa. Therefore, this study sought to address these gaps by sharing our experience among children with MCDK seen at a tertiary paediatric specialist hospital in southern Africa.

## Methods

This retrospective study evaluated all children (< 13 years) diagnosed with MCDK by either antenatal or postnatal abdominopelvic ultrasound, for whom a corresponding confirmatory Mercaptotriglycine ([^99m^Tc]Tc-MAG3) scan was performed at the Red Cross War Memorial Children’s Hospital (RCWMCH) in Cape Town, South Africa, over ten years from 1st January 2014 to 31st December 2023.

At the RCWMCH, children presenting with suspected MCDK, dysplastic cystic kidneys, unilateral renal agenesis, abdominal masses, hydronephrosis, suspected or confirmed urinary tract infection are typically referred for specialist follow-up in the paediatric nephrology clinic. Following an initial (antenatal) or repeat (post-natal) kidney ultrasound, conducted shortly after birth or within the first month of life, a confirmatory [^99m^Tc]Tc-MAG3 scan is routinely performed within six to eight weeks, in accordance with the hospital’s current standard of care, to differentiate a dysplastic kidney with preserved function from a solitary functioning kidney.

Diagnosis of MCDK was based on expanded kidney ultrasound radiological criteria [24], with confirmation of non-functionality established by a [^99m^Tc]Tc-MAG3 scan demonstrating a differential kidney function of 0%. In confirmed cases, serial ultrasound examinations are scheduled at birth, and then at 3, 6, 12, 24, 60, and 120 months. This is done to monitor involution of the MCDK and to assess growth or anomalies of the contralateral kidney. Repeat [^99m^Tc]Tc-MAG3 diuretic scans are reserved for patients exhibiting worsening contralateral hydronephrosis or recurrent urinary tract infections. Surgical intervention is rarely indicated and typically considered only if the dysplastic kidney exceeds 5 cm and increases in size to cause abdominal symptoms, or in cases of recurrent urinary tract infections, unexplained hypertension, or suspected malignancy.

All kidney ultrasound and nuclear medicine scan records are systematically archived in the Picture Archiving and Communication Systems (PACS) and Venus Share Archiving communications system, facilitating digital storage, straightforward retrieval, and comparative referencing with earlier imaging studies.

### Definition of terms

**Vesicoureteral reflux (VUR**) staging on MCUG was classified according to the International Reflux Study Committee Classification [25]. **Ureteropelvic junction obstruction (UPJO)** was defined as the absence of ureter dilatation despite the kidney pelvis exceeding 10–15 mm, calyceal dilatation and an abnormal diuretic renogram pattern. **A duplex system** was defined as a single kidney unit drained by two collecting systems [26]. A unilateral MCDK without genitourinary abnormalities in the bladder or contralateral kidney is a **simple MCDK**, and a unilateral MCDK with genitourinary abnormalities is a **complex MCDK** [19]. **Complete involution** refers to the absence of MCDK evident on the kidney ultrasound. The age at which MCDK becomes undetectable is recognised as the time of complete involution [4]. **Partial involution** indicates a decrease in the size of MCDK, as demonstrated by previous serial kidney ultrasound assessments [4]. **Compensatory hypertrophy of the contralateral kidney** was defined as having a bipolar diameter exceeding two standard deviations above the age-appropriate mean of a normal kidney. In contrast, **the absence of hypertrophy in the contralateral kidney** was indicated by measurements that fall one standard deviation below the mean value of a normal kidney for the corresponding age [27]. **Urinary Tract Infection (UTI)** was characterised by the presence of pyuria (≥ 5 white blood cells per high-power field) and/or a positive leukocyte esterase test or nitrite, along with a positive urine culture. A UTI was diagnosed if a suprapubic bladder aspiration reveals growth of a single bacterium >10^3^ or if a midstream clean-void urine sample shows ≥ 10^5^ colony-forming units/ml in toilet-trained children. Additionally, growth of ≥ 5 × 10^4^ colony-forming units/mL from a transurethral catheterised specimen was also considered a UTI [15]. An **uncomplicated duplex kidney** has two renal moieties with a different pelvicalyceal system, and a **complicated duplex kidney** is one with incomplete duplication, obstructed drainage from a ureterocoele, hydronephrosis, reflux, dysplasia or reduced function [28].

#### Hypertension:

Systolic and/or diastolic blood pressure greater than or equal to the 95th percentile (adjusted for age, gender and height) on at least three different occasions [29]. **Estimated glomerular filtration rate (eGFR)**: The modified bedside Schwartz method was used with the measured height(cm) and serum creatinine [30]. **Proteinuria**: protein creatinine ratio (g/g) on a spot urine sample >0.20 in children over two years of age and >0.50 in infants and toddlers aged 6 to 24 months [31]. **Chronic kidney disease (CKD)** was staged according to KDIGO classification using the eGFR: stage 1 (>90 ml/min/1.73 m^2^ with renal parenchymal damage), stage 2 (90–60 ml/min/1.73 m^2^), stage 3 (60–30 ml/min/1.73 m^2^), stage 4 (30–15 ml/min/1.73 m^2^), or stage 5 (< 15 ml/min/1.73 m^2^) [32]. A child was considered **lost to follow-up (LTFU)** after having consecutively missed at least two years of scheduled follow-up appointments.

### Data collection and flow of eligible children through the study

A comprehensive sampling frame included all eligible children who underwent both [^99m^Tc]Tc-MAG3 and kidney ultrasound scans during the study period, identified from institutional databases. Data were systematically extracted using a standardised proforma from case notes, clinician request forms, and scan reports.

The following variables were collected: patient demographic and anthropometric details at the time of MCDK diagnosis; indication and timing of both KUB and [^99m^Tc]Tc-MAG3 scans; physical examination findings; and serum creatinine levels, where available. Each KUB scan report was reviewed to document the type of MCDK, affected kidney size (mm), number of cysts, anteroposterior diameter (APD), as well as the size (mm), corticomedullary differentiation (CMD), and the presence of a visible ureter in the unaffected kidney. Correspondingly, [^99m^Tc]Tc-MAG3 reports were scrutinised for differential function in each kidney, evidence of obstruction, and confirmation or exclusion of MCDK non-functionality.

Additional data were extracted regarding findings from micturating cystourethrograms (MCUG), which were performed typically following the presence of upper tract dilatation on ultrasound scan or suspected reflux suggested on [^99m^Tc]Tc-MAG3. The presence of concomitant renal or extra-renal anomalies, as well as complications such as hypertension, recurrent urinary tract infections, or malignant transformation, was also recorded. Clinical outcomes were documented, including involution status (complete or partial), kidney function (if recorded during or at the last follow-up visit), management approach (conservative or surgical), requirement for kidney supportive therapy, and final status (ongoing follow-up, transition to adolescent/adult care, loss to follow-up, or death).

Data were analysed using IBM SPSS version 30.0.0. Normality of the collated data was assessed, with continuous variables summarised as means and standard deviations for normally distributed data, or as medians and interquartile ranges (IQR) otherwise; trends were illustrated using line graphs. Categorical variables were presented using frequency tables and charts. Seventeen children who underwent baseline KUB and [^99m^Tc]Tc-MAG3 imaging within their first year of life but lacked at least one year of subsequent follow-up were excluded from longitudinal analyses, and missing data were appropriately addressed.

Predictors of MCDK involution were initially explored using the Log Rank (Mantel-Cox) test in bivariate analysis, followed by Cox proportional hazards regression. Cases without evidence of MCDK involution at the end of follow-up were right-censored. In the multivariate regression, ‘MCDK involuted’ served as the status (dependent) variable and ‘year of involution’ as the time variable. Predictor variables included initial size of the multicystic dysplastic kidney (< 5 cm vs > 5 cm), type of MCDK (simple vs complex), gender, presence of contralateral hypertrophy, presence of contralateral abnormality, and laterality; all were entered as categorical covariates in the Cox model using the ‘Enter’ method. Statistical significance was set at *p* < 0.05. Data were stored in a password-protected Excel file accessible only to the primary researcher, with the patient identification key maintained separately to ensure confidentiality. Each participant was assigned a unique study number, and no identifying information was included in any research outputs.

### Flow diagram of enrolled patients


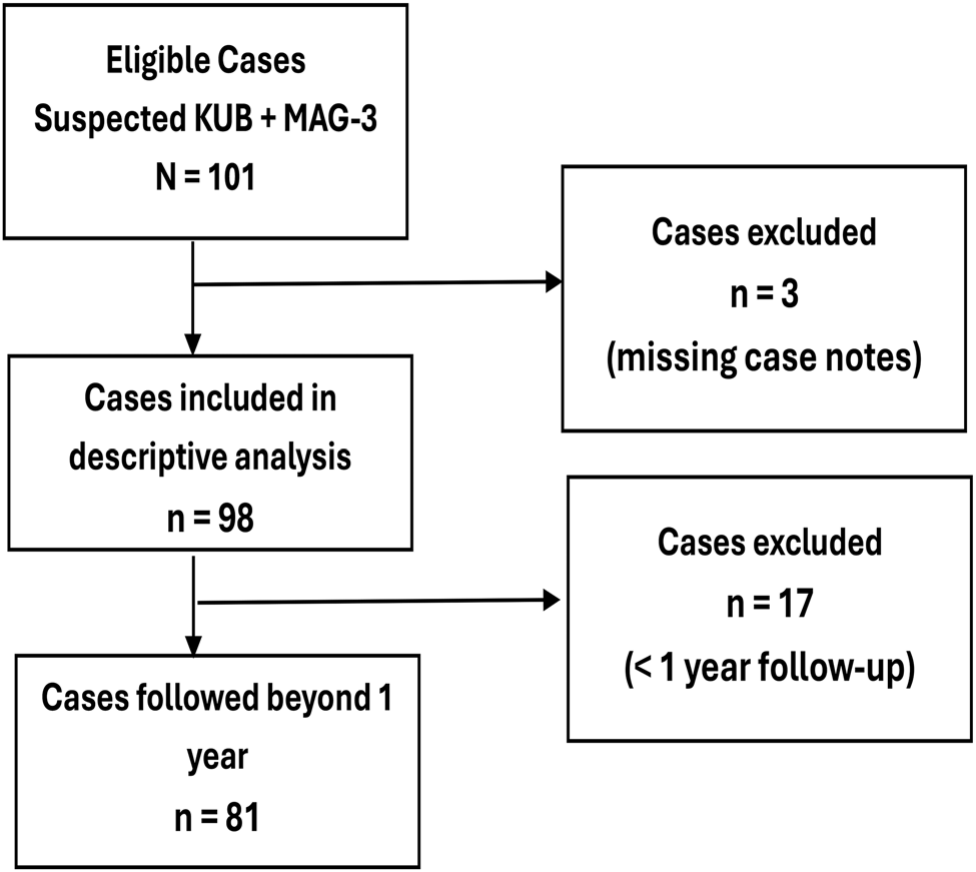


## Results

### Incidence of MCDK among children seen in the renal clinic of RCWMCH

**Fig. 1:**
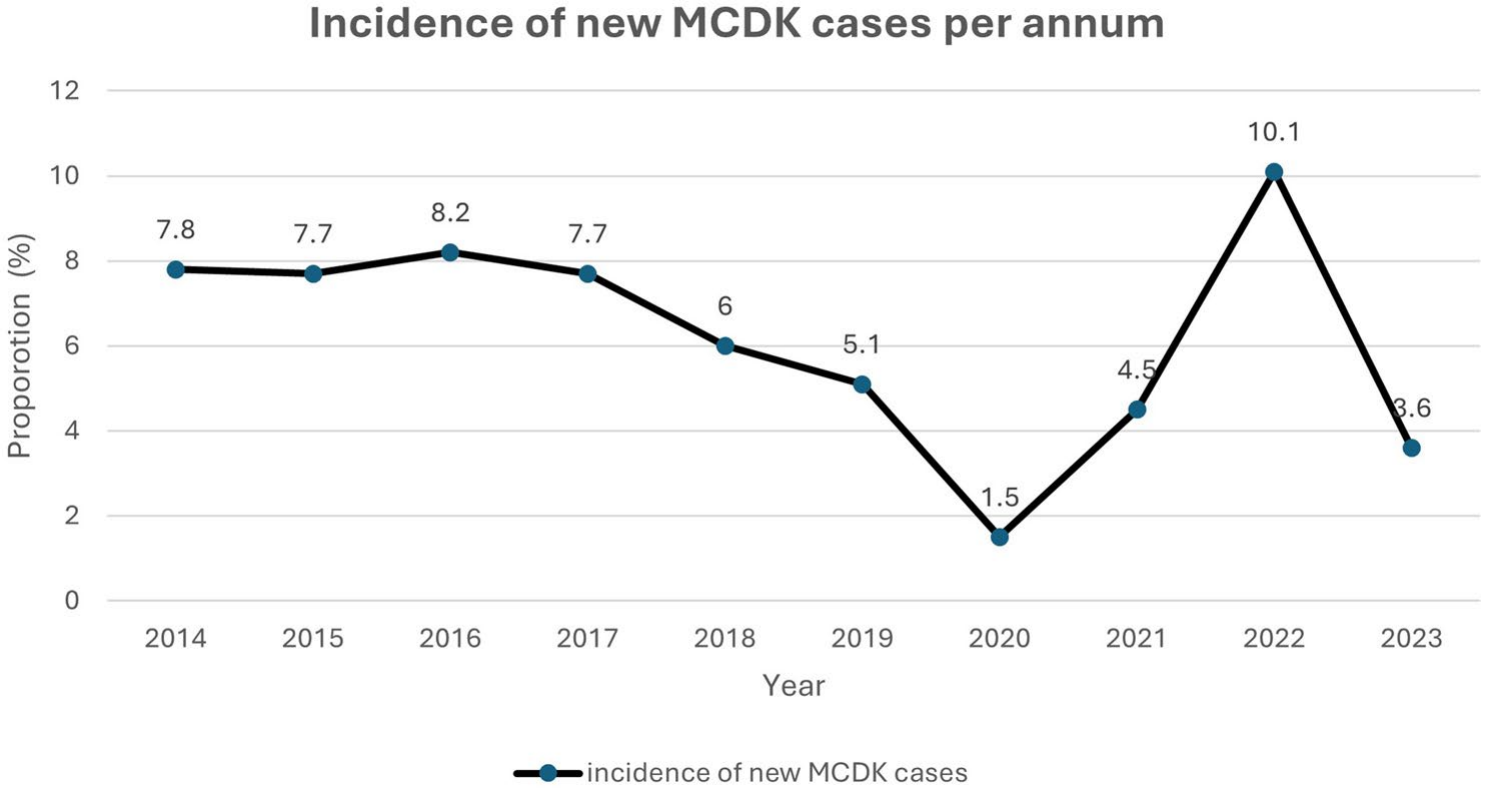
Incidence of MCDK among children seen in the renal clinic

Between January 1, 2014, and December 31, 2023, there were 20,569 paediatric renal outpatient clinic visits, including 1,581 new cases. Of these, 101 children were diagnosed with multicystic dysplastic kidney (MCDK) based on postnatal KUB and [^99m^Tc]Tc-MAG3 scans; three cases were excluded due to unavailable records, resulting in 98 confirmed cases with an MCDK incidence of 6.2%. The incidence varied annually, with the lowest rate of 1.5% observed in 2020 and the highest of 10.1% in 2022, as depicted in Fig. 1.

### Demographic characteristics of study participants

Of the 98 children diagnosed with unilateral MCDK, 84 (85.7%) were identified antenatally, with Groote Schuur Hospital accounting for 60% of referrals. The cohort had a balanced sex distribution, 49 (50.0%) male, and left-sided involvement in 56 (57.1%). The median age at first renal clinic visit was 1.5 months (IQR 1–2) for kidney ultrasound and 2 months (IQR 1.5–3) for [^99m^Tc]Tc-MAG3 scan. Median follow-up was 60 months (IQR 12–72), and median age at last visit was 63.5 months (IQR 41–97). All patients exhibited a non-functioning kidney on [^99m^Tc]Tc-MAG3, with preserved function in the contralateral kidney.

### Patterns of associated genitourinary and extrarenal anomalies among children with MCDK (Table 1)

Contralateral kidney abnormalities were observed in 17 children (17.3%), with duplex kidney being the most common anomaly (35.3%), frequently complicated by hydronephrosis or hydroureters. Other contralateral anomalies included ureteropelvic junction obstruction (29.4%), hydroureteronephrosis and hydronephrosis (each 11.1%) and contralateral dysplasia (11.8%). Ipsilateral hydroureters and ureterocoeles were identified in 4.1% of cases. Additionally, 8.2% of children had extra-renal anomalies, equally divided between syndromic and non-syndromic forms.

**Table 1:** Distribution of abnormalities of the contralateral kidney on ultrasound and associated extrarenal anomalies

	Frequency (*n* = 17/98)	Percent (%)
**Associated anomalies**	17	17.3
**Duplex**	6	35.3
Uncomplicated	2	
Complicated	4	
**UPJO**	5	29.4
**Urinary tract dilatation** Hydroureteronephrosis	4 2	23.5
Hydronephrosis	2	
**Dysplasia**	2	11.8
**Associated abnormalities of the ipsilateral kidney on ultrasound (*n* = 4/98**,** 4.0%)**		
Ureterocoele in the bladder	2	2.0
Hydroureter	2	2.0
**Extrarenal abnormalities (*n* = 8/98**,** 8.2%)**		
**Syndromic (** ***n*** ** = 4)**		
Costello syndrome	1	1.0
4q deletion syndrome	1	1.0
Alagille’s syndrome	1	1.0
Trisomy 21 with ASD	1	1.0
**Non-syndromic (** ***n*** ** = 4)**		
Coarctation of Aorta + Cardiomyopathy	1	1.0
Hydrocolpos	1	1.0
Hemivertebrae (L4/L5)	1	1.0
Isolated oesophageal atresia	1	1.0

UPJO – ureteropelvic junction obstruction

### Estimated glomerular filtration rates, urine and radiology investigations of the study cohort

Of the entire cohort, 81/98 were followed up beyond the first year. Of these, 63 (69.8%) had simple unilateral MCDK, while 18 (25.9%) had complex MCDK. As shown in Tables 2, 15 (18.5%) developed UTIs. One case of UTI was confirmed in the neonatal period. During the later follow-up, 14 children (17.3%) experienced UTIs. The baseline median glomerular filtration rate (GFR) was 62 ml/min/1.73 m² (IQR 35–82), which increased to 121 ml/min/1.73 m² (IQR 90–147) at the last follow-up. Of the 14 children with a recorded eGFR at the final visit, five exhibited hyperfiltration, seven had normal GFRs, and two had persistently reduced GFR (< 90 ml/min/1.73 m²). MCUG were performed in four patients presenting with hydronephrosis, hydroureters, or suspected reflux based on contralateral kidney renogram findings. Of these, one had a dysplastic kidney and three had duplex kidneys; none demonstrated reflux on MCUG.

**Table 2:** Serum creatinine, eGFR, urine and serial radiological investigation history of the study cohort

**Parameter**	**Median (IQR)**			
Median serum creatinine (µmol/L) at baseline (*n* = 21/81)	35.0 (24.5, 64.5)			
Median serum creatinine (µmol/L) at last visit (*n* = 14/81)	33.5 (27.2, 40.25)			
Median eGFR (ml/min/1.73m^2^) at baseline (*n* = 21/81)	62 (35, 82)			
Median eGFR (ml/min/1.73m^2^) at last visit (*n* = 14/81)	121 (90, 147)			
	**Frequency**		**Percent**	
**Urinary Tract Infections** (*n* = 81):	15		18.5	
**Imaging**				
MCUG with no vesicoureteric reflux seen	4		100.0	
**Changes in KUB characteristic findings** **(N = number of children with KUB reports at year of follow-up)**	**Baseline** *N* = 81	**1st year** *N* = 81	**2nd year** *N* = 64	**5th year** *N* = 31
Size of affected kidney (cm)	4.9 (3.6–5.95)	4.1 (2.82–6.2)	4.3 (2.0–7.0)	2.0 (0.0–4.0)
Size of contralateral kidney (cm)	5.5 (5.0–6.1)	6.8 (6.3–7.5)	7.6 (7.3–8.2)	9.0 (8.58–9.60)

### Compensatory hypertrophy of the contralateral kidney

**Fig. 2:**
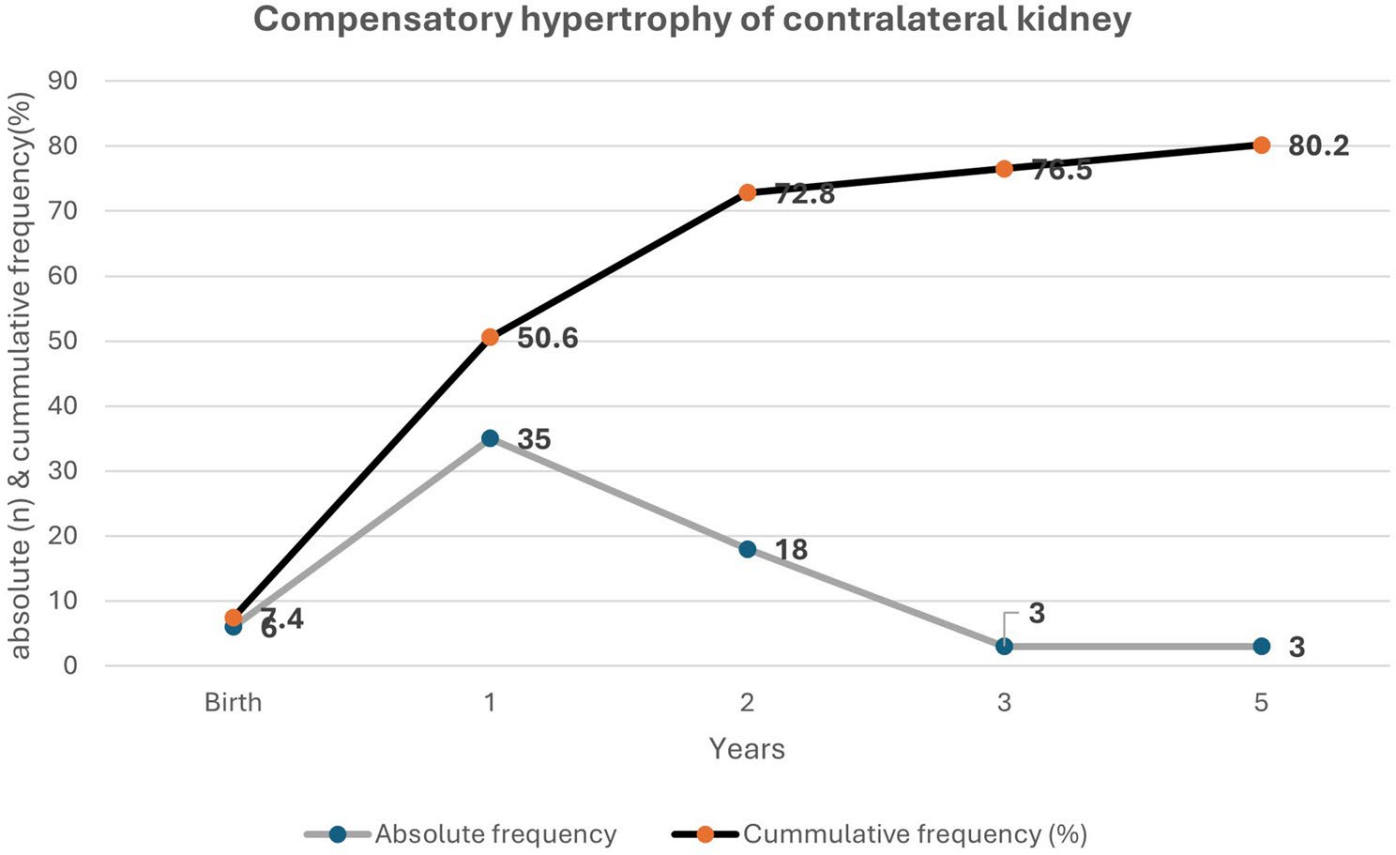
Cumulative frequency of compensatory hypertrophy per year during follow-up

Compensatory hypertrophy of the contralateral kidney was observed in 80.2% of children, with prevalence rising from 7.4% at birth to 80.2% by five years, as shown in Fig. 2.

#### Complications of MCDK among the follow-up cohort of children

During follow-up, one child (1.2%) developed hypertension, and two children developed chronic kidney disease (eGFR < 90 ml/min/1.73 m²); all had contralateral kidney dysplasia. Additionally, two children exhibited persistent sub-nephrotic proteinuria: one with a duplex kidney and upper tract dilatation, and another with contralateral UPJO and 4q deletion syndrome.

### Involution of the MCDK

**Fig. 3:**
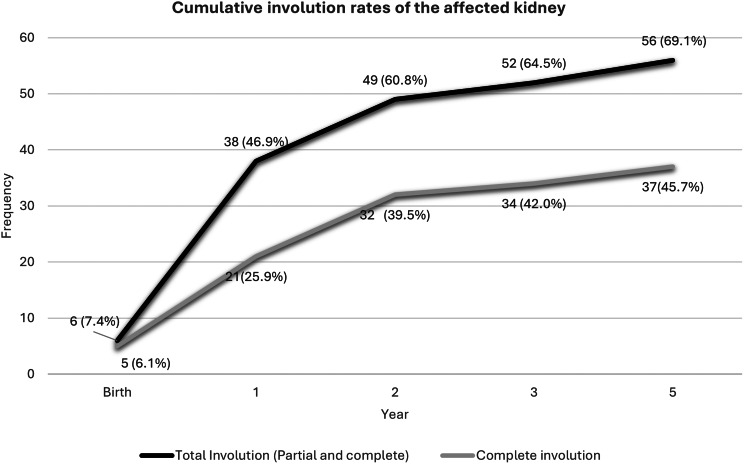
Cumulative involution rates of the affected kidney

Fifty-six children (69.1%) exhibited partial or complete regression of the affected kidney. Complete involution was observed at birth in five cases (6.1%), with cumulative rates of 25.9% at one year, 39.5% at two years, and 45.7% at five years, as illustrated in Fig. 3. Among the 25 children (30.9%) whose kidneys did not involute, 44.0% showed an increase in size, and 56.0% remained stable. The mean age at involution for kidneys ≤ 5.0 cm at baseline was 1.5 ± 1.3 years, compared to 2.0 ± 1.4 years for those > 5.0 cm. The average initial size was 3.6 ± 1.4 cm in children who involuted, versus 6.0 ± 1.9 cm in those who did not.

### Predictors of MCDK Involution

The Log-Rank (Mantel-Cox) test and Cox proportional hazards regression were employed to identify predictors of MCDK involution, as detailed in Table 3. At the bivariate level, both initial MCDK size (*p* < 0.001) and compensatory hypertrophy of the contralateral kidney (*p* = 0.03) were significant predictors. The multivariate Cox regression model, which satisfied the proportional hazards assumption and demonstrated good fit (-2 Log Likelihood = 445.452; omnibus χ² (6) = 13.290, *p* = 0.039), revealed that only initial kidney size remained significant (*p* = 0.005; HR = 2.425, 95% CI: 1.311–4.485) for predicting involution. Specifically, children with an initial MCDK size of ≤ 5.0 cm were more than twice as likely to undergo involution over time compared to those with larger initial sizes. Covariate coding and hazard ratios are presented in Table 3.

**Table 3:** Predictors of Involution of MCDK

	Involution present	Involution absent	Bivariate analysis	Multivariate analysis	
			Log-rank (Mantel-Cox) *p*-value	Cox proportional regression	
				*p*-value	HR (95% CI)
**Type of MCDK**			0.626	0.831	0.930 (0.478–1.809)
Simple	44 (69.8%)	19 (30.2%)			
Complex	12 (66.7%)	6 (33.3%)			
**Gender**			0.330	0.539	0.840 (0.481–1.466)
Male	30 (75.0%)	10 (25.0%)			
Female	26 (63.4%)	15 (36.6%)			
**Compensatory hypertrophy**			0.030*	0.057	2.181 (0.977–4.867)
Yes	49 (75.4%)	16 (24.6%)			
No	7 (43.8%)	9 (56.3%)			
**Mean Kidney size (initial)**	3.6 ± 1.4	6.0 ± 1.9	< 0.001*	< 0.001	0.970 (0.954–0.986) **
**Kidney Size (≤ 5.0 cm)**			0.002*	0.005	2.425 (1.311–4.485) **
Yes	39 (81.3%)	9 (18.8%)			
No	17 (51.5%)	16 (48.5%)			
**Time at diagnosis**			0.422	0.222	1.474 (0.791–2.746)
Antenatal	41 (71.9%)	16 (28.1%)			
Postnatal	15 (62.5%)	9 (37.5%)			
**Contralateral abnormality**			0.626	0.831	0.930 (0.478–1.809)
Yes	12 (66.7%)	6 (33.3%)			
No	44 (68.9%)	19 (30.2%)			
**Laterality of MCDK**			0.346	0.899	1.037 (0.589–1.825)
Right	23 (63.9%)	13 (36.1%)			
Left	33 (73.3%)	12 (26.7%)			

* / ** statistically significant in bivariate and multivariate analysis

t-test statistic 7.1652 mean difference: -2.40; (95% CI; − 3.067 to – 1.733)

#### Surgical and follow-up outcomes

During follow-up, three children (3.7%) required surgery; one nephrectomy for persistently increasing MCDK > 10 cm with intra-abdominal compromise and two had pyeloplasty for contralateral obstruction. At the last clinic visit, median time of 5 years, 56.8% (46) remained under follow-up, 2.5% (2) were transferred out, 12.3% (10) had transitioned to adolescent care, and 28.4% (23) were lost to follow-up. No malignancies or MCDK-related deaths were observed.

## Discussion

This study described the epidemiology of paediatric MCDK as seen in a tertiary specialist renal outpatient clinic in sub-Saharan Africa. MCDK, typically identified in either the perinatal or postnatal period, has a reported incidence of 1 in 2,200 to 4,300 live births [2, 3]. However, these estimates were derived exclusively from data outside sub-Saharan Africa. The apparent underdiagnosis of MCDK in this region may plausibly reflect both limited detection and genetic variability. In our cohort, MCDK accounted for approximately 1 in 16 new renal cases seen at the outpatient clinic during the study period, making it the most common cystic dysplastic kidney disease observed. By comparison, an earlier study by Lala [19] identified 59 cases among 10,445 admissions over 30 years in Johannesburg, corresponding to a prevalence of 5.7 per 1,000 admissions, although differences in denominators limit direct comparison. Notably, our findings demonstrate an increasing incidence of MCDK over the past decade, which may be attributable to enhanced expertise in feto-maternal ultrasonography, improvements in ultrasound technology, and our role as a regional referral centre. Accurately assessing the incidence of MCDK is challenging due to its often asymptomatic presentation until later life [33]. In South Africa, the lack of multicentre or nationwide registries for CAKUT surveillance precludes reliable regional estimates. Furthermore, the observed reduction in incidence in 2020 is most likely a consequence of reduced healthcare access during the COVID-19 pandemic.

The predominance of prenatal ultrasound diagnoses in this cohort signifies progress in early MCDK detection relative to an earlier South African finding [19]. Referrals primarily originated from tertiary centres, indicating improved diagnostic precision attributable to technological advancements and consensus guidelines. Most cases were identified during third-trimester scans, consistent with reports from Spain [34] and a recent meta-analysis by Erlich and colleagues [35]. Furthermore, contemporary studies demonstrate that prenatal diagnostic accuracy for MCDK has reached rates as high as 91.3% – 98.0% [14, 33, 36, 37], underscoring the increasing effectiveness of prenatal ultrasound in this context.

Approximately one-fifth of children in this cohort exhibited contralateral kidney abnormalities, consistent with the reported range of 5% – 43% in other studies [21], though lower than the 33% pooled estimate from the meta-analysis by Schreuder and colleagues.^3^ Vesicoureteric reflux (VUR) is frequently cited as the most common abnormality, with pooled rates of 17% – 20% [3, 35]. However, it was not reflected in our cohort. Notably, mild reflux was detected by [^99m^Tc]Tc-MAG3 renogram in a small number of cases, but not confirmed by MCUG, as previously observed in South African studies [19, 38]. This finding underpins the selective use of MCUG, reserving it for cases with contralateral or bladder abnormalities on kidney ultrasound scan, rather than routine application in all children with MCDK.

Our study found duplex kidney to be the most common contralateral abnormality, observed in one-third of affected cases, a finding scarcely reported in other studies. Particularly, kidney duplication was not identified as an abnormality in earlier systematic reviews [3, 35], with only sporadic reports documenting such findings [39, 40].

Ureteropelvic junction obstruction was the second most frequent contralateral anomaly, with a subset of patients requiring surgical intervention to preserve kidney function. Ipsilateral abnormalities were rare, affecting less than a tenth of the cohort, markedly lower than the prevalence reported in other studies [33, 41, 42]. The primary ipsilateral findings were ureterocoeles and hydroureters, whereas previous studies have also described vesicoureteral reflux and internal genital tract anomalies [41, 42].

Consistent with the literature, this study found that approximately 1 in 10 children with MCDK had extra-renal anomalies, including rare syndromic associations suggestive of a possible genetic component in some cases [43–46]. Although the prevalence of extrarenal associations in our cohort was lower than previously reported rates of 25–48% [37, 47] these findings emphasise the importance of thorough physical examination in children with MCDK.

Compensatory hypertrophy of the contralateral kidney is well-documented in MCDK and is associated with involution of the affected kidney after two years of age [48]. In this study, compensatory hypertrophy was observed in less than one-tenth of children at birth, increasing to four-fifths by five years, consistent with previous reports of rapid early growth in the functional kidney [5, 9, 14, 15, 49–51]. However, about one-fifth of our cohort did not develop compensatory hypertrophy, highlighting the need for ongoing surveillance, as the absence of hypertrophy may indicate underlying kidney dysfunction [27].

Studies have reported on the involution rates of MCDK over time, with approximately 5–6% showing complete involution at birth [3], a finding mirrored in this study. Our cohort demonstrated cumulative involution probabilities, aligning with international data [4, 22, 34, 52, 53]. Variations in involution rates across studies may reflect differences in follow-up duration, sample size, and scan quality. Consistent with earlier studies, an initial MCDK size of ≤ 5 cm was found to be a positive predictor of complete involution [4, 8, 53].

At presentation, most children had low eGFRs, reflecting immature kidney function due to young age. Only a subset of patients had follow-up eGFR measured, with median values increasing to normal levels and some demonstrating hyperfiltration without proteinuria. Routine kidney function was not a standard of care in our setting if there were no suspected risks, like recurrent UTIs or contralateral abnormalities. Two children developed CKD (eGFRs < 90 ml/min/1.73 m²) due to renal dysplasia of the contralateral kidney. The first case had coexisting hydronephrosis with recurrent UTIs. The second was a syndromic child (4q deletion) who also had UPJO. Neither of them showed involution of the multicystic dysplastic kidney. The first experienced a symptomatic UTI with acute kidney injury in the first year of presentation. The second exhibited elevated creatinine due to PUJO, with some improvement after pyeloplasty. At their last follow-up, the eGFRs corresponded to CKD stages 2 and 4, respectively. The latter also had sub-nephrotic proteinuria and developed elevated blood pressure by age ten.

Our findings align with previous studies highlighting variability in kidney function and proteinuria among children with MCDK [22, 53, 54]. Furthermore, our findings suggest the need for baseline and at least annual monitoring of kidney function and proteinuria to detect early signs of kidney injury, regardless of perceived risk. Given the potential for hyperfiltration and the implications of Brenner’s and Barker’s hypotheses, children with MCDK, particularly those with low birth weight or complex type disease, may require long-term surveillance and lifestyle counselling to mitigate future cardiometabolic and chronic kidney risks [9, 55].

At the last follow-up clinic visit, most children with MCDK remained under surveillance, while a proportion had been transferred to adolescent care or to facilities outside Cape Town. Notably, nearly one-quarter of the cohort were lost to follow-up, a trend potentially attributed to caregiver reassurance following favourable prognoses, a phenomenon previously observed in another study [56]. This is concerning, given the enduring risk of chronic kidney disease, as evidenced by the Solitary Functioning Kidney: Aetiology and Prognosis (SOFIA) study, which reported that a substantial proportion of patients with a solitary functioning kidney (SFK) exhibited markers of kidney injury by late adolescence [11]. Even in children with unilateral MCDK and a normal contralateral kidney, the risk of developing stage II CKD remains appreciable by age ten [50, 55, 57]. These findings highlight the necessity of thorough education for caregivers regarding long-term renal risks and highlight the importance of implementing strategies to improve follow-up adherence.

No cases of malignancy, mortality, or need for kidney replacement therapy were reported over the study period. Conservative management, involving serial ultrasonography until spontaneous involution, proved effective, offering reduced procedural risks and financial savings, as similarly noted in earlier studies [12, 21]. The optimal frequency of kidney ultrasound scan monitoring remains contentious, with protocols ranging from intensive early follow-up to interval-based surveillance at key developmental milestones [16, 22, 54, 55, 58]. In light of this cohort’s largely benign short-term outcomes, adopting a less intensive follow-up regimen, including kidney ultrasound scans at birth, one month, two years, five years, and ten years, respectively, as suggested by Hains et al. [21] and Aslam et al. [22], appears appropriate for the present study’s setting. We suggest this approach because our protocol of a more frequent follow-up regimen did not necessarily improve clinical follow-up compliance.

Therefore, this study demonstrates that paediatric MCDK can be accurately diagnosed, monitored, and managed conservatively in resource-constrained settings. Moreover, reducing the number of clinic appointments could enhance patient compliance with follow-up visits and alleviate pressure on the already overburdened healthcare system.

This single-centre study was constrained by its retrospective design, with some incomplete or missing data, variability in the documentation of clinical notes and laboratory test requests, which could potentially impact the reliability and generalisability of the findings and introduce possible bias.

## Conclusion

Children with MCDK in South Africa experience favourable short-term outcomes, particularly in the absence of contralateral abnormalities. The duplex kidney and ureteropelvic junction obstruction were the most common contralateral kidney abnormalities. Most children exhibited compensatory hypertrophy by age 2, suggesting good adaptation, with gradual changes observed until age 5, which supports the recommendation of limiting ultrasound frequency. Follow-up into adolescence may be beneficial, as progression of chronic kidney disease is rare in those without contralateral anomalies. Multicentre long-term studies are needed to provide standardised follow-up guidelines.

### Take-home messages

- In children with MCDK, conservative management is sufficient, and there is no need for nephrectomy.
- Frequency of kidney ultrasound scan monitoring at birth, 1 month, 2 years, 5 years, and 10 years (depending on resource availability) is a suggested approach.
- A yearly follow-up kidney health assessment – including blood pressure and urine dipstick checks, with reassessments every one or two years, is a reasonable approach.

## Data Availability

The data that support the findings of this study are available from the corresponding author and Dr Coetzee, of the department of paediatrics, nephrology division, RCWMCH, but restrictions apply to the availability of these data, which were used under license for the current study, and so are not publicly available. Data are, however, available from the authors upon reasonable request and with permission of the Ethical Approval Committee of the Institution.

## Abbreviations

CI: Confidence interval
CKD: Chronic kidney disease
eGFR: Estimated glomerular filtration rate
HR: Hazard ratio
IQR: Interquartile range
KDIGO: Kidney Disease Improving Global Outcomes
KUB Scan: Kidney Ureter Bladder Ultrasound Scan
[^99m^Tc]Tc-MAG3: ^99m^Tc Mercaptoacetyltriglycine-3
MCDK: Multicystic dysplastic kidney
MCUG: Micturating Cystourethrogram
RCWMCH: Red Cross War Memorial Children’s Hospital
SFK: Solitary functioning kidney
UPJO: Ureteropelvic junction obstruction
UTIs: Urinary tract infections
VUR: Vesicoureteric reflux
